# Association between Anaemia, Iron Deficiency Anaemia, Neglected Parasitic Infections and Socioeconomic Factors in Rural Children of West Malaysia

**DOI:** 10.1371/journal.pntd.0001550

**Published:** 2012-03-06

**Authors:** Romano Ngui, Yvonne Ai Lian Lim, Liam Chong Kin, Chow Sek Chuen, Shukri Jaffar

**Affiliations:** 1 Department of Parasitology, Faculty of Medicine, University of Malaya, Kuala Lumpur, Malaysia; 2 Department of Medicine, Faculty of Medicine, University of Malaya, Kuala Lumpur, Malaysia; 3 School of Science, Monash University, Jalan Lagoon Selatan, Selangor Darul Ehsan, Malaysia; University of Kelaniya, Sri Lanka

## Abstract

**Background:**

Given that micronutrient deficiency, neglected intestinal parasitic infections (IPIs) and poor socioeconomic status are closely linked, we conducted a cross-sectional study to assess the relationship between IPIs and nutritional status of children living in remote and rural areas in West Malaysia.

**Methods/Findings:**

A total of 550 children participated, comprising 520 (94.5%) school children aged 7 to 12 years old, 30 (5.5%) young children aged 1 to 6 years old, 254 (46.2%) boys and 296 (53.8%) girls. Of the 550 children, 26.2% were anaemic, 54.9% iron deficient and 16.9% had iron deficiency anaemia (IDA). The overall prevalence of helminths was 76.5% comprising *Trichuris trichiura* (71.5%), *Ascaris lumbricoides* (41.6%) and hookworm infection (13.5%). It was observed that iron deficiency was significantly higher in girls (p = 0.032) compared to boys. Univariate analysis demonstrated that low level of mother's education (OR = 2.52; 95% CI = 1.38–4.60; p = 0.002), non working parents (OR = 2.18; 95% CI = 2.06–2.31; p = 0.013), low household income (OR = 2.02; 95% CI = 1.14–3.59; p = 0.015), *T. trichiura* (OR = 2.15; 95% CI = 1.21–3.81; p = 0.008) and *A. lumbricoides* infections (OR = 1.63; 95% CI = 1.04–2.55; p = 0.032) were significantly associated with the high prevalence of IDA. Multivariate analysis confirmed that low level of mother's education (OR = 1.48; 95 CI% = 1.33–2.58; p<0.001) was a significant predictor for IDA in these children.

**Conclusion:**

It is crucial that a comprehensive primary health care programme for these communities that includes periodic de-worming, nutrition supplement, improved household economy, education, sanitation status and personal hygiene are taken into consideration to improve the nutritional status of these children.

## Author Summary

Micronutrient deficiency and intestinal parasitic infections (IPIs) share a similar geographical distribution. A conservative estimate indicated that almost 2 billion individuals suffer from anaemia due to iron deficiency (ID), corresponding to 24.8% of the world's population. Crucially, most of these individuals are children and women of reproductive age in developing countries. Intestinal parasitic infections, especially soil-transmitted helminthes (STH), are prevalent in areas where micronutrient deficiency is widespread and the relationship between them has been studied. Most studies have noted an association between iron deficiency and IPIs. Against this background we studied the association between micronutrient deficiency, IPIs and socioeconomic factors among rural children in West Malaysia. Overall, 26.2%, 54.9% and 16.9% of the participants had anaemia, ID and iron deficiency anaemia (IDA), respectively. The overall prevalence of STH infections was 76.5% with *Trichuris trichiura* (71.5%), *Ascaris lumbricoides* (41.6%) and hookworm (13.5%). Univariate analysis found that low level of mother's education, i.e., less than 6 years of formal education, non working parents, low house income, *T. trichiura* infection and *A. lumbricoides* infection were significantly associated with the odds of IDA. The final multivariate analysis indicated that low level of mother's education was a significant predictor for IDA in these children.

## Introduction

Anaemia is a specific condition where red blood cells are not providing adequate oxygen to the body tissues. It is usually caused by iron deficiency, which is the commonest micronutrient deficiency in both developing and developed countries [1], [2]. Generally, it takes at least several weeks after iron store has depleted before anaemia develops. When iron deficient occurs, haemoglobin concentrations are reduced to below optimal levels and therefore, iron deficiency anemia (IDA) is considered to be present. However, because anaemia is the most common indicator used to screen for iron deficiency, the terms anaemia, iron deficiency, and IDA are sometimes used interchangeably and synonymously [2].

Group most affected include pregnant women, pre-school and school-age children, low birth weight infants and women of child-bearing age [1], [3]. The World Health Organization (WHO) estimates that more than two billion people are affected by iron deficiency and anaemeia, which corresponds to 24.8% of the world's population [1]. Most are in the Western Pacific and South-East Asia. Despite its increasing prevalence in South-East Asia, anaemia is the most neglected nutritional deficiency disorder in the region today [4]. Iron deficiency anaemia (IDA) has severe nutritional and health consequences, including inadequate growth and mental development in children [5], high maternal mortality and incidence of low birth weight infants and low productivity in adults [5], [6]. Poor school performance among school children and adolescent has also been associated with IDA [7], [8].

Micronutrient deficiency causes are multifactorial ranging from micronutrient deficiency such as iron, folate and vitamin B_12_, insufficient dietary intake, malabsorption and infectious diseases in particular parasitic infections [9]. The latter is well documented and soil-transmitted helminth (STH) infections are prevalent in areas where anaemia and IDA is widespread [10]. Accumulating evidence from a number of studies has shown that micronutrient deficiency and STH infections are intertwined and co-exist among low-income population [11], [12]. Other determinants such as demographic factors such as age, gender and larger family size [13], [14], and low educational attainment of parents [15]–[17] has shown to have significant association with both anaemia and IDA, particularly among the rural and poor communities in developing countries.

In Malaysia, the impact of STH infections on nutrition, growth and development have been studied [11], [12], [16], [18], [19]. Malnutrition is often associated with IDA because of the low intake of heme iron from animal food sources, derived from low quality diet because of poverty. Very often malnourished individuals are also anaemic and they are often associated with high parasitic infections, particularly STH infections and malaria. Several studies have demonstrated that STH infections are strong indicators of malnutrition such as IDA and low serum retinol (i.e., indicative of vitamin A deficiency) [19]–[21]. Given that the nutritional deficiency, neglected intestinal parasitic infections and poor socioeconomic status are closely linked, we conducted a comprehensive study to provide current information on a continuing problem on the prevalence of anaemia, iron deficiency, IDA, intestinal parasitic infections and also to investigate their possible associated factors among 550 children living in remote and rural areas in West Malaysia.

## Methods

### Study Areas and Subjects

A cross-sectional study was carried out between November 2007 to July 2009 among 550 children living in 8 villages from 5 different states in remote and rural areas of West Malaysia. The villages were selected based on (i) village entry approval by the Ministry of Rural and Regional Development Malaysia, (ii) STH infections, anaemia and IDA are known to be high and (iii) it is accessible by road transportation for rapid transfer of samples to the laboratory. Each village had a small population, and the number of children in each village was estimated to be between 20 to 100. Majority of the parents (71.8%; 409/570) did not have any formal education. More than half (85.6%) of the parents of the children did odd jobs such as selling forest products without any stable income. Some were daily wage earners working in rubber or palm oil plantations, unskilled laborers in factories or construction sites. Therefore, more than half (76.3%) of the households which the children belonged to earned less than RM 500 per month (<US$ 166.7), the poverty income threshold in Malaysia [22] which is inadequate to maintain a good living standard.

Although majority of the children's houses have provision of basic infrastructure such as treated water supply (56.5%), at least 43.5% are still using untreated water originating from a nearby river for their domestic needs. All children that agreed voluntarily to participate were included in this study. The inclusion criteria were children below 12 years old, residence in rural and remote areas and provided consent to participate. Exclusion criteria included having medical condition for which follow-up was required or refusal to participate. Although 700 questionnaires and faecal containers were distributed, only 550 stool and blood samples were collected the following day resulting in a response rate of 78.6%. Hence, 150 (21.4%) children who did not turn up or failed to provide their faecal or blood samples were excluded from the study.

### Questionnaire Survey

An interview-administrated questionnaire was used to elicit and gather information from participants on the demographic (i.e., age, gender and education attainment), socioeconomic (i.e., father or mother occupation, household income) and medical treatment (i.e., whether the participant has taken anthelminthic drugs and iron supplement) of the participants which will assist the assessment of potential factors associated with serum iron status. It was adapted from a standard questionnaire that was previously used in recent studies on IPIs and iron serum indicator in Malaysia [11]. The questionnaire was first designed in English and then translated and pretested in Malay language, which is the national language for Malaysia and well understood by the participants. For very young children, the questionnaire was completed by interviewing their parents and guardians or the relevant adult (normally head of the family) who signed the informed consent. Participants who participated in this study were honored with a small token of appreciation.

### Blood Collection and Determination of Iron Status

Approximately 5 ml of venous blood sample was drawn from each qualified participant who fulfilled the specific criteria by trained medical assistants and nurses. Each blood sample collected was distributed into 2 different tubes: (i) Becton Dickinson Vacutainer K2 EDTA tube (anticoagulant ethylene diamine tetraacetic chloride) and (ii) plain tube (without anticoagulant). The blood samples were kept in standard storage box with ice pack and transported back to the Department of Parasitology, Faculty of Medicine, University of Malaya for further analysis. Blood samples were spun at 1500 rpm for 10 minutes and the sera and plasma were kept at −20°C until use. All the samples were analyzed within 3 to 10 hours after blood collection.

Iron status was determined by measuring the haemoglobin, serum ferritin (SF) and serum iron (SI) levels [3]. Individuals are categorized as iron deficient when the SF level falls below the cut-off value (see below). For IDA, individuals should be both anaemic (low Hb level) and iron deficient (low SF level with or without low SI level). Hb was measured using an automated hematology cell counter analyzer (Sysmex XE-2100, Sysmex America, Inc.). The procedure was carried according to manufacturer's guidelines. Anaemia was confirmed when the Hb level is <110 g/L for children aged 6 to 59 months, <115 g/L for children 5 to 11 years, <120 g/L for children 12 years [2].

The level of SF was determined using the ADVIA Centaur System Assay (Bayer ADVIA Centaur; Siemens Healthcare Diagnostics, NY, USA). This is a two-site sandwich immunoassay using direct chemiluminometric technology, which uses constant amounts of two anti-ferritin antibodies. The procedure was carried out according to the ADVIA Centaur Assay Manual (111653 Rev. G, 2003–2005) as provided by the manufacturer's guidelines. Iron deficiency (ID) was determined when SF levels are <15 µg/L for individuals aged >4 years. In addition, a lower cut-off value (<10 µg/L) was also used for participants 1 year old [2].

SI was determined calorimetrically using the FerroZine method on the COBAS INTEGRA 400/800 (Iron Gen.2) analyzer (Roche Diagnostic GmbH, Indianapolis, IN, USA). The procedures were carried out according to the manufacturer's guidelines. Individuals with concentrations below the cut-off value (<11 µmol/L) were considered to have ID [2]. Individuals with normal Hb levels and low SF level with or without low SI level were classified as iron deficiency. All these procedures pertaining to Hb, SF and SI were carried out at the Clinical Diagnostic Laboratory, University Malaya Medical Centre (Malaysia).

### Faecal Collection and Detection of Parasites

For the examination of parasites in faeces, a wide mouth and screw capped faecal container with an attached scoop were labeled, coded and distributed to each participant together with plastic bag after the questionnaires were completed. The participant was instructed to scoop a thumb size faecal sample using a provided scoop into the container, making sure that the sample was not contaminated with urine for collection on the following day. The collected faecal samples were processed using formalin ether concentration technique [23] followed by iodine staining and microscopy examination for the presence of STH infections. In addition, Kato-Katz technique was employed to determine the intensity of STH infections, as estimated by egg counts per gram of faeces for the presence of *Ascaris lumbricoides*, *Trichuris trichiura* and hookworm ova [24]. The worm burden was classified as light, moderate and heavy based on the threshold proposed by WHO Expert Committee [25]. Dysenteric or inadequate samples, which were unsuitable for egg counts were used only for the confirmation of the presence of STH ova by formalin ether concentration technique.

### Statistical Analysis

Statistical analysis was carried out using the SPSS software (Statistical Package for the Social Sciences) programme for windows version 13 (SPSS, Chicago, IL, USA). For descriptive data, percentage rate was used to describe the characteristics of the studied population, including the prevalence of anaemia, IDA and STH infections. The distribution of Hb, SF and SI were presented as median and inter quartile range (IQR) after being examined for normality using the Kolmogrov-Smirnov Z test. The intensity of STH infections (worm burden) was quantitatively estimated as ova per gram of faeces and was categorized into three main categories: light, moderate and heavy infections. The distribution of egg counts for all the three STH species were not normally distributed, hence presented as median.

Crude associations of the binary outcome variable (for proportion) were assessed by Pearson's Chi-square (*X^2^*). For each categorical variable, odds ratios (ORs) and 95% confidence interval (95% CI) were calculated using univariate analysis. As for the continuous variables, i.e., Hb, SI, SF value and egg count of the three STH species were not normally distributed, therefore the correlation between each of these variables were computed using Spearman's correlation coefficients (r_s_) test. The association between anaemia, iron deficiency and IDA and their determinants were examined by univariate and multivariate logistic regression analysis. Significant variables in univariate analysis (p<0.05) were included in a logistic multivariate analysis (stepwise regression) to determine which factors could be dropped from the multivariable model. The level of statistical significance was set as p<0.05 and for each statistically significant factor, an odds ratio (OR) and 95% confidence interval (95% CI) were used for all test to explore the strength of the association between anaemia, ID and IDA and the variable of interest.

### Ethical Consideration

The study protocol was approved and granted by the Ethics Committee of the University Malaya Medical Centre (MEC Ref. No. 638.36). Prior to participation, surveyors introduced themselves and an oral briefing to describe the objective and methodology of the study was given to the participants. They were also informed of the potential risk of employed procedures and the assurance that their identity and personal particulars will be kept confidential and anonymously. Participation was voluntary and the children could withdraw from the study at any time without giving any reason. Consent of those who agreed to participate were taken either in written form (signed) or verbally followed by their thumb prints and from parents or guardians (on behalf of the very young children).

## Results

### Characteristics of the Study Population

A total of 550 children (254 boys and 296 girls) were recruited in this study. With regards to age groups, there were a total of 30 (5.5%) young children aged 1 to 6 years and 520 (94.5%) school children aged 7 to 12 years with a median age of 10 years.

### Prevalence of Anaemia, Iron Deficiency and IDA

The present study showed that 26.2% (144/550) of the participants had anaemia, 54.9% (302/550) had ID and 16.9% (93/550) had IDA (Table 1). The median concentration of haemoglobin, serum ferritin and serum iron was 126.0 g/l (IQR = 119.0–133.0), 26.1 µg/l (IQR = 13.0–51.6) and 11.5 µmol/l (IQR = 8.4–15.3), respectively. The prevalence of iron status was further analyzed according to gender and age groups (Table 1). Generally, the occurrence of anaemia, ID and IDA were not significantly associated with age and gender. Although the prevalence of anaemia in young children aged 1 to 6 years (36.7%) was higher compared to school children aged 7 to 12 years (25.6%), but the differences was not statistically significant. Similarly, there was no significant difference between ID and IDA with age groups although the prevalence of ID and IDA were high among young children compared to school children. With regards to gender, it was observed that only ID was significantly higher in girls (X^2^ = 4.50; p = 0.032) compared to boys.

**Table 1:** Distribution of serum iron status among rural children in West Malaysia (N = 550)^a^.

		Hb (g/l)	SF (µg/l)	SI (µmol/l)	Anaemia^b^		ID^c^		IDA^d^	
Age/Gender	N	Median (IQR)	Median (IQR)	Median (IQR)	No.	%	No.	%	No.	%
**Age (year)**										
1–6	30	121.0 (109.0–131.0)	24.5 (10.9–48.4)	10.5 (5.1–14.8)	11	36.7	17	56.7	8	26.7
7–12	520	126.0 (119.0–133.0)	26.2 (13.2–52.2)	12.0 (8.3–15.1)	133	25.6	285	54.8	85	16.3
**Gender**										
Boy	254	126.0 (120.0–133.0)	29.9 (14.3–53.5)	11.5 (8.4–15.3)	61	24.0	127	50.0	40	15.7
Girl	296	126.0 (118.0–133.0)	23.6 (12.1–48.5)	11.7 (8.5–15.5)	83	28.0	175	59.1*	53	17.9
**Total**	**550**	**126.0 (119.0–133.0)**	**26.1 (13.0–51.6)**	**11.5 (8.4–15.3)**	**144**	**26.2**	**302**	**54.9**	**93**	**16.9**

N: number examined; IQR: Inter quartile range.

a Distribution of anaemia, iron deficiency (ID) and iron deficiency anaemia (IDA) according to gender and age groups.

b Haemoglobin (Hb) below age-specific threshold (see methodology).

c Serum ferritin (SF) <15 µg/l with or without serum iron (SI) <11 µmol/l.

d Presence of both anaemia and iron deficiency (ID).

*: Significant difference (p<0.05).

### Prevalence and Intensity of Soil Transmitted Helminth (STH) Infections

The overall prevalence of STH infections was 76.5% (421/550). Among all the three species of STH infections, *Trichuris trichiura* (71.5%; 393/550) was the most predominant, followed by *Ascaris lumbricoides* (41.6%; 229/550) while only 13.5% (74/550) had hookworm infections (data not shown). Off these, double infections (36.5%; 201/550) was most common followed by single infection (33.3%; 183/550) and 6.7% (37/550) were infected with all three worm infections. The combination of *T. trichiura* and *A. lumbricoides* (31.8%; 175; 550) was the most common, followed by *T. trichiura* and hookworm (3.1%; 17/550) and hookworm and *A. lumbricoides* (1.6%; 9/550). As for single infection, *T. trichiuria* infection (29.8%; 164/550) was the most prevalent, followed by hookworm infection (2.0%; 11/550) and *A. lumbricoides* (1.5%; 8/550) (data not shown).

With regards to the intensity of infections, not all microscopically positive samples were examined by Kato Katz due to the limited amount of sample available. This resulted in only 391 out of 393 microscopically positive for *T. trichiura*, 224 out of 229 for *A. lumbricoides* and 65 out of 74 for hookworm were examined by Kato Katz, respectively (Table 2). The median egg counts among infected children were 1,465 (range: 22–115,085 epg) for *T. trichiura*, 6,638 (range: 22–365,190 epg) for *A. lumbricoides* and 44 (range: 22–4,129 epg) for hookworm infection.

**Table 2:** Intensity of STH infections among rural children in West Malaysia.

	Type of infections								
Intensity of infections	*T. trichiura* (N = 391)			*A. lumbricoides* (N = 224)			Hookworm (N = 65)		
	no.	%	Median	no.	%	Median	no.	%	Median
Light	142	36.3	511	92	41.7	2,231	46	70.8	22
Moderate	207	52.9	2,353	124	55.4	12,565	18	27.7	163
Heavy	42	10.7	14,896	8	3.6	56,521	1	1.5	NA

N: Number examined; no: Number positive; NA: Not available; EPG: Eggs per gram.

The proportion of the infected children was further explored according to intensity of infection for all the three STH species (Table 2). The mean egg count for hookworm showed a significant difference with age (F = 0.034; p<0.001) as determined by one way ANOVA of log 10 transformed egg count. However, the mean egg count for *T. trichiura* and *A. lumbricoides* did not show any significant variation with age. Similarly, there was no significant difference between mean egg counts of all the three STH species with gender (data not shown).

### Relationship between STH Infections, Anaemia, Iron Deficiency (ID) and IDA

The prevalence of *T. trichiura* and *A. lumbricoides* infections were found to be significantly associated with anaemia and IDA. However, no prevalence of any STH infections was significantly associated with ID. There was a significant correlation between increasing *T. trichiura* egg counts and decreasing Hb value, however, this correlation was weak (r_s_ = −0.122; p = 0.016). As for *A. lumbricoides* and hookworm egg counts, no significant correlation was found with Hb value. Likewise, the SI value was also not significantly correlated with any of the three STH species egg counts. With regards to SF values, *A. lumbricoides* egg count was significantly correlated with SF values (r_s_ = −0.150; p = 0.019).

The distribution of iron status indicator was also analyzed according to intensity of STH infections (Table 3). The Hb, SF and SI levels declined from light infection to heavy infection for *T. trichiura*, *A. lumbricoides* and hookworm infections. Although the prevalence of anaemia, ID and IDA increased with increasing worm burden, it was not statistically correlated with any of the worm burden thresholds.

**Table 3:** Distribution of anaemia, iron deficiency and iron deficiency anaemia according to intensity of STH infections.

Type of		Hb	SF	SI	Anaemia^a^		ID^b^		IDA^c^	
infections	N	(g/l)	(µg/l)	(µmol/l)	No.	%	No.	%	No.	%
***T. trichiuria***										
Light	142	126.0	37.3	11.8	36	25.3	79	55.6	23	16.2
Moderate	207	124.0	38.2	11.8	65	31.4	114	55.1	42	20.3
Heavy	42	122.0	36.5	10.9	15	35.7	26	61.9	12	28.6
***A. lumbricoides***										
Light	92	126.0	36.7	13.8	23	25.0	53	57.6	13	14.1
Moderate	124	124.0	37.3	11.1	45	36.3	79	63.7	32	25.8
Heavy	8	123.0	33.1	11.8	2	25.0	3	37.5	2	25.0
**Hookworm**										
Light	46	123.0	48.1	10.1	17	38.9	33	71.7	13	28.2
Moderate	18	120.0	25.6	12.7	7	36.9	7	38.9	3	16.6
Heavy	1	112.0^d^	4.0^d^	5.0^d^	1	100	1	100	1	100

a Haemoglobin (Hb) below age-specific threshold (see methodology).

b Serum ferritin (SF) <15 µg/l with or without serum iron (SI) <11 µmol/l.

c Presence of both anaemia and iron deficiency (ID).

d Value was based on single infected individual.

### Aetiological Factors Associated with IDA

The association of IDA in relation to IPIs and sociodemographic characteristics were examined by univariate analysis (Table 4). The results showed that low level of mother's education, i.e., less than 6 years of formal education (OR = 2.52; 95% CI = 1.38–4.60; p = 0.002), non working parents (OR = 2.18; 95% CI = 2.06–2.31; p = 0.013), low house income (OR = 2.02; 95% CI = 1.14–3.59; p = 0.015), *T. trichiura* infection (OR = 2.15; 95% CI = 1.21–3.81; p = 0.008) and *A. lumbricoides* infection (OR = 1.63; 95% CI = 1.04–2.55; p = 0.032) were significantly associated with the odds of IDA.

**Table 4:** Odds ratios of the aetiological factors for IDA among 550 rural children in West Malaysia^a^.

	Normal	IDA	Crude	
Variable	(n = 457)	(n = 93)	OR (95% CI)	P value
Age group 1–6 years	4.8	8.6	1.79 (0.82–3.89)	0.143
Female	52.5	57.6	1.07 (0.88–1.30)	0.501
Mother's educational level (<6 years of formal education)	69.1	84.9	2.52 (1.38–4.60)	0.002^b^ *
Non working parents	71.3	83.9	2.18 (2.06–2.31)	0.013^b^
Low household income (RM <500 per month/<US$ 166.7)	70.5	82.8	2.02 (1.14–3.59)	0.015^b^
Not receiving any iron supplement in the last 12 months	18.3	23.9	1.40 (0.79–2.48)	0.244
Not receiving any anthelminthic drug in the last 12 months	41.1	48.4	1.17 (0.93–1.50)	0.197
Any *T. trichiura* infection	69.1	82.8	2.15 (1.21–3.81)	0.008^b^
Any *A. lumbricoides* infection	39.6	51.6	1.63 (1.04–2.55)	0.032^b^
Any hookworm infection	12.7	17.2	1.43 (0.78–2.62)	0.245
*T. trichiura* and *A. lumbricoides* infections	30.9	36.6	1.30 (0.81–2.06)	0.282
*T. trichiura* and hookworm infections	2.8	4.3	1.54 (0.49–4.82)	0.459
*A. lumbricoides* and hookworm infections	1.5	2.2	1.41 (0.29–6.91)	0.668
*T. trichiura*, *A. lumbricoides* and hookworm infections	5.9	10.8	1.92 (0.90–4.11)	0.089
Severe trichuriasis (epg >10 000)	9.6	15.6	1.75 (0.85–3.60)	0.126
Severe ascariasis (epg >50 000)	3.4	4.3	1.27 (0.25–6.49)	0.776
Severe hookworm (epg >4000)	0.0	5.9	c	c

a Odds ratios of the aetiological factors for IDA among 550 rural children in West Malaysia with corresponding 95% confidence intervals (95% CI) and P values resulting from logistic univariate and multivariate stepwise regression.

b Variables were included in the logistic multivariate analysis.

c Cornfield 95% CI for odd ratio is not accurate due to low numbers.

*: Significant variable by multivariate logistic regression.

A logistic regression model was used to assess the effects of the significant explanatory variables in order to distinguish predictors of IDA. The final multivariate analysis indicated that only low level of mother's education, i.e., less than 6 years of formal education (OR = 1.48; 95 CI% = 1.33–2.58; p<0.001) was a significant predictor for IDA in these children.

## Discussion

Anaemia is regarded worldwide as a medical condition deserving of sustained public health intervention and still a major public health problem in many developing countries, especially in rural communities. It is estimated that most children and preganant women in developing and 40.0% in developed countries are iron deficient [2]. Findings of the present study demonstrated that the overall prevalence of anaemia was 26.2% while 54.9% had ID and 16.9% had IDA among rural and remote children of West Malaysia. This is in concordance with a study among rural adolescents in Sabah (East Malaysia), which found that the prevalence of anaemia and IDA was 20.0% and 17.0%, respectively [13]. However higher rates were reported in other local studies. The most recent study conducted among rural school children in Malaysia reported a prevalence of 48.5% for anaemia and 34.0% for IDA [11]. Similarly, another local study which has been conducted among rural children documented 41.5% and 36.0% of anaemia and IDA, respectively [16].

When compared to data from other countries, the present results demonstrated that the prevalence of anaemia and IDA among rural children in Malaysia was relatively higher. In south-eastern Brazil, the prevalence of anaemia, ID and IDA was 11.8%, 12.7% and 4.3%, respectively among populations living in highly endemic area of hookworm infection [26] and relatively lower prevalence of anaemia (16.5%) was also reported in South Africa [27]. However, anaemia was extremely high (92.0%) in Kenya, which could be attributed to the high prevalence of malaria in the study area [28]. Nevertheless, findings of this present study are parallel with the most recent study carried out among Nigerian children where 38.6% children were found to be anaemic [29]. Similarly, study in northeast Thailand also reported 31.0% of the children to be anaemic [30].

Among the age groups, the prevalence of anaemia, ID and IDA were higher among young children compared to school children. However, this finding could be attributed to the benefit of school children having access to iron supplementation (sponsored by the government) in rural schools. The present study found low prevalence of IDA among children who received iron supplement within the last 12 months although it was not statistically significant. A local study investigated the effects of iron-folate supplements administered at school and it was found that iron-folate supplementation has a direct benefit in improving iron nutrition on these schoolchildren [31]. It has also been demonstrated that this is a practical, safe, effective and inexpensive method for improving the wellbeing of school children [31].

The low numbers of young children, i.e., less than 6 years old who participated in the present study compared to school children has unable us to investigate the casual association between cases of anaemia, ID and IDA with age groups. Nonetheless, previous studies conducted in Malaysia [11], [16], Thailand [17] and Brazil [26] have documented that cases of anaemia decreased with age. The poor daily iron intake together with poverty and infections could be the main factors contributing to high prevalence of anaemia and IDA among the children involved in this study. Although efforts to obtain meaningful dietary assessment was unsuccessful in this study, other studies have shown that daily iron intake among rural children was low and inadequate, achieving only 29.0% to 49.0% of the Recommended Daily Intake (RDI) [11].

In the present study, 76.5% of the children were infected with at least one of the STH species. *T. trichiura* (71.5%) infections proved to be the most common compared to *A. lumbricoides* (41.6%) and hookworm (13.5%). This is in agreement with other previous local studies where *T. trichiura* infections had the highest prevalence (range: 26% to 98.2%), followed by *A. lumbricoides* infections (range: 19% to 67.8%) and lastly hookworm infections (range: 3% to 37%) [16], [32]–[35]. Therefore, the present findings not only showed that the prevalence of STH remains high but the trend of distribution of STH also remains unchanged in these rural children [34]. The higher rate of STH infection especially *T. trichiura* infection could be due to the ineffective dosage and choice of anthelminthic used or drug resistance and has been discussed in our previous study [35].

The aetiology of anaemia and IDA and the reasons for its ubiquitous persistence are multi-factorial and complex [1], [2]. Interactions of many factors that co-exist such as poor dietary intake, increased demands (e.g., growth), parasitic infections, socioeconomic causes and genetic factors (e.g., thalassaemia) may be causes of anaemia and IDA. Therefore, the present study also highlighted and assessed the relationship between the associated factors underlying iron status. There was significant association between anaemia and IDA in those who were infected with *T. trichiura* compared to uninfected individuals as reported in the present study. This is in line with other studies where *T. trichiura* infection is a significant predictor for anaemia and IDA among Panamanian [7] and Kenyan children [36]. The present study also demonstrated that those infected with severe *T. trichiura* were almost two times more likely to suffer from IDA, which is similar to previous studies where high intensity of *T. trichiura* infection as a significant risk factor for anaemia and IDA [11], [16]. Studies conducted in south-eastern Brazil [26] and East Africa [37] also showed significant association between intensity of *T. trichiura* and hookworm infections with anaemia and IDA.

In *T. trichiura* infections, it is well accepted that the infection may involve significant blood loss given the location of the worm in the large intestine [7]. Blood depletion is even worse in cases where trichuriasis is in concomitant with hookworm infections. Adult hookworms tend to inhabit the upper small intestine, whereas mature *T. trichiura* inhabit the upper caecum and colon. Bleeding due to hookworm infection occurs in the upper small intestine and some of the constituents from the blood are reabsorbed further down in the gastrointestinal tract. Therefore, it is possible that the re-absorption of iron may be impaired, either by ingestion of the iron by *T. trichiura* or by the mal-absorptive surface of the gut [7]. Moreover, severe *T. trichiura* infection also causes colitis leading to dysentery and chronic faecal blood loss.

In our study, we also found significant relationship between *A. lumbricoides* infections and IDA among these rural children. Similarly, a most recent study conducted among rural Nigerian children found significant association between *A. lumbricoides* infection and anaemia [29]. Likewise, study among Zanzibari schoolchildren also demonstrated that an *A. lumbricoides* infection was associated with lower Hb values [38]. Iron is absorbed through the intestinal wall in the duodenum and jejunum and it is believed that iron absorption could be impaired by the presence of *A. lumbricoides* in this part of the intestine [39].

Although we found no significant association between hookworm infection nor intensity of the infection with iron status, the prevalence of anaemia and IDA were higher among those infected with hookworm infections. This finding is parallel with a study conducted among Vietnamese [40] and Ugandan [41] children where no association was established between hookworm infection and anaemia but disagrees with a most recent study among children living in rural Nigeria [29]. This is also in contrast with studies conducted in East Africa which found that iron stores depleted even with light eggs counts [37]. Additionally, previous evidences have demonstrated that hookworm infection as an important aetiological cause of anaemia and IDA among infected individuals [26], [36]–[38]. This could be due to a low prevalence of hookworm among the children, whom mainly had low infection intensity. It is also possible that the hookworm infection among the children in the present study were too light to have significant impact on their iron status. It is also likely that iron stores were not sufficiently depleted for hookworm to be associated with anaemia.

The present study also highlighted the impact of low socioeconomic status on their iron status among these rural children. In addition, parental educational attainment (especially mothers) also plays an importance role on the health of the children as demonstrated in the present study whereby children of parent with low educational background are more likely to develop IDA than children of parent with higher educational background. Similar observation has been reported in study among rural children in Malaysia [11]. Likewise, study conducted in Brazil also reported the low level of Hb and SF among children of illiterate parent [42]. Significant association between non working parents and IDA was also noted in the present study, which corroborated with previous study, showing significant correlation between IDA and non working parent in India [15]. However, there is a disparity between the present findings with a previous study, which found a significant association between IDA and working parent [11], [16]. Low household income (<RM 500 per month/<US$ 166.7) was also significantly associated with the high prevalent of IDA in the present study. Previous study also attributed poverty, unavailability of nutritious food and proper health care as significant contributing factors [11]. Such an association between low household income and malnutrition was also reported among rural children in Malaysia [43], [44]. A study in Thailand observed that there is a significant increase in risk of acquiring IDA when household income decreases [17].

The present study has several limitations. Firstly, the findings were based only on single point data collection, i.e., cross-sectional study, and may therefore fail to identify direct casual association between IDA and it determinants. The casual associations between IDA and risk factors of importance can be confirmed by pre and post intervention observational studies that compare the effects of deworming treatment on iron status. There have been numerous evidences that showed regular antihelminthic treatment does improve the iron status of the infected individuals. Study conducted to evaluate the effects of school based deworming program on iron status among children in Zanzibar [45] and Tanzania [46] demonstrated significant improvement of the Hb and serum ferritin concentrations after treatment with anthelminthic drug.

Secondly, the results on IDA which depended solely on SF as the indicator for iron status in these populations burdened with high prevalence of infections may lead to underestimation of iron deficiency. Serum ferritin, a reputedly more stable marker of iron status has been used widely as a reliable index in nutritional survey and clinical assessment for the replacement of SI and total iron binding capacity (TIBC) [47], however, many studies have demonstrated that SF tends to be elevated during the presence of acute or chronic inflammation [48]. It is well known that infections can lead to inflammation and SF level may often reach up to 20 µg/L in the present of inflammation even in the presence of marked iron deficiency [49].

Unfortunately, we have not been able to consider the effect of inflammation on the assessment of SF given that not all infected individual with intestinal parasites had inflammation and vice versa. Assumption of the presence of inflammation in all subjects or those infected individual could lead to underestimation of the number of cases with low SF. Therefore, it is highly recommended to use different indicators such as C-reactive protein (CRP) or α-1 chymotrypsin (ACT) to distinguish between inflamed and non-inflamed individuals in indicating whether a normal or high level of ferritin can truly represent adequate iron stores in future study [49]. With regards to this, we have tried our best to minimize the effect of inflammation on SI level such as by excluding any children with evidence of severe and chronic inflammation from our study and also by using a low cut-off point for SF (<10 µg/L) so that there can be little doubt that iron status was deficient. Nevertheless, despite all these limitations, this study is one of the few quantitative, comprehensively analyzed studies on the epidemiology of parasitic infection, iron status and its determinants among Malaysian children.

In conclusion, our results provided a comprehensive current population-based iron status among children living in highly endemic area of IPIs coupled with poor socioeconomic background. As the alleviation of poverty among rural communities is critical as illustrated by the health consequences, some of the major issues which propagate poverty such as being left out of the country's mainstream development need to be address urgently and holistically. It is crucial that a comprehensive primary health care programme for these communities which includes periodic de-worming, nutrition supplement, improved household economy, education, sanitation status and personal hygiene are taken into consideration to improve the nutritional status of these children.

## Supporting Information

Checklist S1 STROBE checklist.
(DOC)

## References

[1] de Benoist B, McLean E, Egli I, Cogswell M, WHO (2008). Worldwide prevalence of anaemia 1993–2005.. WHO global database on anaemia.

[2] WHO (2001). Iron deficiency anaemia. Assessment, prevention, and control. A guide for programme managers.

[3] Mei Z, Cogswell ME, Parvanta I, Lynch S, Beard JL (2005). Hemoglobin and ferritin are currently the most efficient indicators of population response to iron interventions: An analysis of nine randomized controlled trials.. J Nutr.

[4] Gopalan C (1992). Strategies for combating under nutrition: lessons learned for the future. In: Nutrition in developmental transition in Southeast Asia.

[5] Crompton DWT, Stephenson LS, Schad GA, Warren KS (1990). Hookworm infection, nutritional status and productivity.. Hookworm disease: Current status and new directions.

[6] Larocque R, Casapia M, Gotuzzo E, Gyorkos TW (2005). Relationship between intensity of soil-transmitted helminth infections and anemia during pregnancy.. Am J Trop Med Hyg.

[7] Robertson LJ, Crompton DW, Sanjur D, Nesheim MC (1992). Haemoglobin concentrations and concomitant infections of hookworm and *Trichuris trichiura* in Panamanian primary schoolchildren.. Trans R Soc Trop Med Hyg.

[8] Seshadri S, Gopaldas T (1989). Impact of iron supplementation on cognitive functions in preschool and school-aged children: the Indian experience.. Am J Clin Nutr.

[9] Yip R, Ramakrishnan R (2002). Experiences and Challenges in developing countries.. J Nutri.

[10] Foy H, Nelson GS (1963). Helminths in the etiology of anaemia in the tropics, with special reference to hookworms and schistosomes.. Exp Parasitol.

[11] Al-Mekhlafi HMS, Johari S, Atiya AS, Ariffin WA, Mohammed Mahdy AK (2008). Anaemia and iron deficiency anaemia among aboriginal schoolchildren in rural Peninsular Malaysia: An update on a continuing problem.. Trans Roy Soc Trop Med Hyg.

[12] Khor GK, Zalilah MS (2008). The ecology of health and nutrition of Orang Asli (Indigenous people) women and children in Peninsular Malaysia.. Tribes and Tribals.

[13] Foo LH, Khor GL, Tee ES (2004). Iron status and dietary iron intake of adolescents from a rural community in Sabah, Malaysia.. Asia Pac J Clin Nutr.

[14] Mai TT, Hung NK, Kawakami M, Kawase M, Chuyen NV (2003). Micronutrient status of primary school girls in rural and urban areas of South Vietnam.. Asia Pac J Clin Nutr.

[15] Sinha N, Deshmukh PR, Garg BS (2008). Epidemiology correlates of nutritional anaemia among children (6–35 months) in rural Wardha, Central India.. Indian J Med Sci.

[16] Nor Aini U, Al-Mekhlafi HM, Azlin M, Shaik A, Sa'iah A (2007). Serum iron status in Orang Asli children living in endemic areas of soil-transmitted helminthes.. Asia Pac J Clin Nutr.

[17] Issaragrisil S, Kaufman DW, Anderson TE, Chansung K, Thamprasit T (1995). An association of aplastic anaemia in Thailand with low socioeconomic status.. Br J Haematol.

[18] Zulkifli A, Khairul AA, Atiya AS, Yano A (1999). Malnutrition and helminth infections among pre-school children in Orang Asli resettlement villages in Kelantan.. JUMMEC.

[19] Al-Mekhlafi MH, Azlin M, Aini UN, Shaik A, Sa'iah A (2007). Prevalence and predictors of low serum retinol and hypoalbuminaemia among children in rural Peninsular Malaysia.. Trans R Soc Trop Med Hyg.

[20] Curtale F, Pokhrel RP, Tilden RL, Higashi G (1995). Intestinal helminthes and xerophthalmia in Nepal: a case-control study.. J Trop Pediatr.

[21] Muniz-Junqueira MI, Queiroz EF (2002). Relationship between protein-energy malnutrition, vitamin A and parasitoses in children living in Brasilia, Brazil.. Rev Soc Bras Med Trop.

[22] Department of Statistics Malaysia (1997). Profile of Orang Asli in Peninsular Malaysia..

[23] Allen AVH, Ridley DS (1970). Further observations on the formol-ether concentration technique for faecal parasites.. J Clin Pathol.

[24] Martin LK, Beaver PC (1968). Evaluation of Kato thick smears technique for quantitative diagnosis of helminth infections.. Am J Trop Med Hyg.

[25] Montresor A, Cromptom DWT, Bundy DAP, Hall A, Savioli L (1998). Guidelines for the evaluation of soil-transmitted helmintiasis and schistomiasis at community level.. WHO/CTD/SIP.

[26] Brooker S, Jardim-Botelho A, Quinnell RJ, Geiger SM, Caldas RS (2008). Age-related changes in hookworm infection, anaemia and iron deficiency in an area of high *Necator americanus* hookworm transmission in south-eastern Brazil.. Trans R Soc Trop Med Hyg.

[27] Janabhai CC, Taylor M, Coutsoudis A, Coovadia HM (2001). A health and nutritional profile of rural school children in Kwazulu Natal, South Africa.. Ann Trop Paediatr.

[28] Nabakwe EC, Lichtenbelt W, Ngare DK (2005). Vitamin A deficiency and anaemia in young children living in a malaria endemic district of western Kenya.. East Afr Med J.

[29] Osazuwa F, Ayo OM, Imade P (2011). A significant association between intestinal helminth infection and anaemia burden in children in rural communities of Edo state, Nigeria.. North Am J Med Sci.

[30] Thurlow RA, Winichagoon P, Green T, Wasantwisut E, Pongcharoen T (2005). Only a small proportion of anemia in northeast Thai schoolchildren is associated with iron deficiency.. Am J Clin Nutr.

[31] Tee ES, Kandiah M, Awin N, Chong SM, Satgunasingam N (1999). School-administered weekly iron-folate supplements improve hemoglobin and ferritin concentrations in Malaysian adolescent girls.. Am J Clin Nutr.

[32] Hakim SL, Gan CC, Malkit K, Azian MN, Chong CK (2007). Parasitic infections among Orang Asli (aborigine) in the Cameron Highlands, Malaysia.. Southeast Asian J Trop Med Pub Health.

[33] Rajeswari B, Sinniah B, Hussein H (1994). Socioeconomic factor associate with intestinal parasites among children living in Gombak, Malaysia.. Asia Pac J Clin Nutr.

[34] Lim YAL, Romano N, Colin N, Chow SC, Smith HV (2009). Intestinal parasitic infections amongst Orang Asli (indigenous) in Malaysia: Has socioeconomic development alleviated the problem?. Trop Biomed.

[35] Ngui R, Saidon I, Chow SC, Rohela M, Lim YAL (2011). Prevalence and risk factors of intestinal parasitism in rural and remote West Malaysia.. PloS Neglected Tropical Disease.

[36] Brooker S, Peshu N, Warn PA, Masobo M, Guyatt H (1999). The epidemiology of hookworm infection and its contribution to anaemia among preschool children on the Kenyan Coast.. Trans R Soc Trop Med Hyg.

[37] Stoltzfus RJ, Chwaya HM, Montresor A, Albonico M, Savioli L (2000). Malaria, hookworms and recent fever are related to anemia and iron status indicators in 0 to 5 year old Zanzibari children and these relationships change with age.. J Nutr.

[38] Stoltzfus RJ, Chwaya HM, Tielsch JM, Schulze KJ, Albonico M (1997). Epidemiology of iron deficiency anemia in Zanzibari schoolchildren: the importance of hookworms.. Am J Clin Nutr.

[39] Islek I, Kuçukoduk S, Cetinkaya F, Gurses N (1993). Effects of *Ascaris* infection on iron absorption in children.. Ann Trop Med Parasitol.

[40] Hung LQ, Giao PT, Peter V, Binh TQ (2005). Anaemia, malaria and hookworm infections in a Vietnamese ethnic minority.. Southeast Asian J Trop Med Pub Health.

[41] Green HK, Sousa-Figueiredo JC, Basanez MG, Betson M, Kabatereine NB (2011). Anaemia in Ugandan preschool-aged children: the relative contribution of intestinal parasites and malaria.. Parasitology.

[42] de Almeida CA, Ricco RG, Ciampo LA, Souza AM, Pinho AP (2004). Factors associated with iron deficiency anemia in Brazilian preschool children.. J Pediatr.

[43] Zamaliah M, Mohd N, Khor G, Siong T (1998). Socio-economic determinants of nutritional status of children in rural Peninsular Malaysia.. Asia Pac J Clin Nutr.

[44] Norhayati M, Noorhayati MI, Mohammod CG, Oothuman P, Azizi O (1997). Malnutrition and its risk factors among children 1–7 years old in rural Malaysian communities.. Asia Pacific J Clin Nutr.

[45] Stoltzfus RJ, Albonico M, Chwaya HM, Tielsch JM, Schulze KJ (1998). Effects of the Zanzibar school-based deworming program on iron status of children.. Am J Clin Nutr.

[46] Bhargava A, Jukes M, Lambo J, Kihamia CM, Lorri W (2003). Anthelmintic treatment improves the hemoglobin and serum ferritin concentrations of Tanzanian schoolchildren.. Food Nutr Bull.

[47] Cook J (1999). The nutritional assessment of iron status.. Arch Latinoam Nutr.

[48] Witte DL (1991). Can serum ferritin be effectively interpreted in the presence of the acute phase response?. Clin Chem.

[49] Tomkins A (2003). Assessing micronutrient status in the presence of inflammation.. J Nutr.

